# Trial for Abortion

**Published:** 1860-07

**Authors:** 


*Trial for Abortion.—The Queen v. White and others.*—Mr. Kennedy applied on Tuesday last, at the Court of Queen's Bench, in this case, for a rule to show cause why the Coroner's proceedings (the latter of two inquisitions) should not be quashed. The two prisoners were White, a chemist at Birmingham, and a Mrs. Fisher, with whom a female had lodged for six or seven weeks; and, after taking certain medicines and undergoing operations at the hands of White, died. At a first inquest a verdict of "Natural Death" was returned; at a second, one of "Wilful Murder" against the prisoners. It was contended that the Coroner had acted wrongly in taking the evidence, on the second occasion, of a witness who had been present and not examined on the first, and on whose statement the case turned. This important application was adjourned to search for precedents.

